# Depressive Symptoms Affect Working Memory in Healthy Older Adult Hispanics

**DOI:** 10.4172/2167-1044.1000204

**Published:** 2015-09-27

**Authors:** Monica Salazar-Villanea, Edward Liebmann, Mauricio Garnier-Villarreal, Esteban Montenegro-Montenegro, David K. Johnson

**Affiliations:** 1 University of Costa Rica Department of Psychology, USA; 2 University of Kansas, Department of Psychology, USA

**Keywords:** Depression affect, Working memory, Latin America, Costa Rica

## Abstract

**Objectives:**

Low and middle income nations will experience an unprecedented growth of the elderly population and subsequent increase in age-related neurological disorders. Worldwide prevalence and incidence of all-types of neurological disorders with serious mental health complications will increase with life expectancy across the globe. One-in- ten individuals over 75 has at least moderate cognitive impairment. Prevalence of cognitive impairment doubles every 5 years thereafter. Latin America’s population of older adult’s 65 years and older is growing rapidly, yet little is known about cognitive aging among healthy older Latinos. Clinically significant depressive symptomatology is common among community-dwelling older adults and is associated with deficits across multiple cognitive domains, however much of the literature has not modeled the unique effects of depression distinct from negative and low positive affect. Our objective was to understand how mental health affects cognitive health in healthy aging Latinos.

**Methods:**

The present study used confirmatory factor analysis (CFA) and structural equation modeling (SEM) to examine the relative effects of Negative Affect, Positive Affect and Geriatric Depression on Verbal Memory, Verbal Reasoning, Processing Speed, and Working Memory in healthy aging Latinos. Data was collected from a sample of healthy community dwelling older adults living in San Jose, Costa Rica. Modeling of latent variables attenuated error and improved measurement reliability of cognition, affect, and depression variables.

**Results:**

Costa Ricans enjoy a notoriety for being much happier than US citizens and are renowned as one of the happiest nations in the world in global surveys. This was born out in these data. Costa Rican affective profiles differed substantively from US profiles. Levels of negative affect and depression were similar to US samples, but their levels of positive affect were much higher. Cognitive performance of these Costa Rican older adults was similar to US-age and education matched peers. CFA and SEM found that increased depressive symptomatology had deleterious effects on Working Memory made up of subtest scores sampling simple attention and vigilance for numbers. Verbal Memory, Verbal Reasoning, and Processing Speed were not affected by self-reported Positive Affect, Negative Affect or Depressive symptoms.

**Conclusion:**

Costa Rican older adults were happy, as evidenced by the high ratio of positive affect to relatively low negative affect. Thus, we were somewhat surprised to find that depressive symptoms were selectively correlated to decrements in working memory and that negative and positive affect contributed negligible amounts of variance to any of the cognitive factors. Because of the methodological rigor of latent variable analysis, these results are very specific. The Working Memory factor is not contaminated with Speed of Processing or other measured cognitive factors. Likewise, the measured Geriatric Depression represents symptoms that are richly cognitive, not overtly affective.

## Introduction

Developing nations have the fastest growing populations of adults 65 and older [1]. Decreasing mortality rates and increasing life expectancies in developing nations will give rise to an older-adult population whose health will be greatly affected by limited education, limited healthcare, malnutrition, and other risk factors. These risk factors are significant public health concerns for the developing world as they are determinants of neurodegenerative disease such as Alzheimer’s disease (AD) and other chronic noncommunicable diseases, which are rapidly growing as the predominant causes of death worldwide [2]. The growth rate of the older-adult population in Latin America is estimated to be particularly great.

In Costa Rica, the population of adults 65 and older is estimated to grow by 262% between 2008 and 2040 compared to 107% in the United States during the same period [1]. This rate is in part attributable to the remarkably low mortality rate of Costa Rican elders, which is lower than the mortality rate in the United States. Unlike the mortality rate in the United States, the Costa Rican morality rate is inversely related to increased SES and education [3]. Many factors likely drive the differential health outcomes between Costa Rica and the United States. Costa Ricans and Americans have differential relationships between education and cardiovascular outcomes, while cognitive status and depression have been positively associated with age and SES in both countries [3,4].

Depression is an important health outcome for older adults (OA) worldwide and associated with increased risk of mortality and dementia [5–8]. The one-year point prevalence from the Cache County study for geriatric major depression (MDD) was 4.4% for women and 2.7% for men [9]. No estimate of late-life depression prevalence exists for Costa Rican OA. The 10/66 study found that the prevalence of late-life depression was 1.3–2.8% across Peru, Mexico, and Venezuela [10]. Estimates of the prevalence of subthreshold depression are 2–3 fold that of depression and have been estimated to account for 10% community dwelling elderly population [11]. Thus, it is likely that subthreshold depression significantly exceeds that of major depression in Latin America and Costa Rica as well. Associations between depression and cognition have been established in studies of Brazilian, Ecuadorean, and Mexican OA [12–14]. We know of no studies conducted in Costa Rica. To understand better the determinants of the unique heath profile of Costa Rican OA, it is important to test the associations of physical, mental and cognitive health outcomes.

### Depression and cognitive impairment

Depression in healthy older adults has been associated with cognitive deficits and longitudinal decline on measures of memory, attention, executive functioning, and everyday problem solving [15–19]. Although the association between depression and cognitive decline is consistent, variability exists in the specific cognitive domains associated with depression. This may be related to sample and methodological characteristics including the measure used to assess depression, current medications/recent changes to medication regimen, physical illness/ functional decline, history of ECT, and whether depression is late-onset or early-onset [20].

The cognitive deficits in executive functioning, episodic memory, and processing speed associated with depression have been demonstrated along the continuum of depression severity [21]. Research has demonstrated that OA with subthreshold depression perform intermediately between healthy controls and better depressed OA, but the reliability of these findings is currently unknown [22,23]. Studies have shown that OA with lower-severity depressive disorders such as dysthymia and minor depression have elevated risk for dementia comparable to OA with major depression at follow-up and have comparable cognitive deficits 12 months post-hospitalization [5,24]. The preliminary research on minor depression and OA suggests that minor depression and major depression more likely fall along a single continuum than represent distinct disorders [25].

### Symptom profile of geriatric depression

Difficulties separating medical conditions from depressive symptomatology and the recognition of novel depressive syndromes in OA, such as vascular depression, have made defining characteristic symptom clusters and individual symptoms of geriatric depression in OA imperative [26,27]. Studies using latent class analysis to identify unique depression symptom clusters for OA have been mixed. Some latent class analyses reproduced the gradient of depression severity (i.e. no depression, mild/moderate, severe) while others derived clusters that differed minimally or were characterized by variability in factors related to depression such as functional impairment, perceived well-being, and perceived social support [27,28]. Other latent class analyses have derived depression clusters with syndromes marked by psychomotor changes, sleep disruption, and fatigue and constellations of other depression symptoms intermediate in severity between non-depressed clusters and the MDD clusters [29]. Recently, researchers have advocated for additional attention to the specific symptoms of geriatric depression [29]. Older adults have been found to report more anhedonia, reduced well-being, more insomnia, more fatigue, slower speed of processing, more thoughts of death/desire to die, more psychomotor retardation, and more somatic symptoms while depressive affect, sadness, lost appetite, and concentration are relatively stable across adulthood [29–32]. Gallo, Anthony, and Muthén [33] found that the anhedonia/dysphoria item on the Diagnostic Interview Schedule (DIS) was less likely to be indicated by adults 65 and older. However, this item blends dysphoria and anhedonia and thus does not likely contradict the finding of other studies of age-related increases in anhedonia.

Some of the symptoms characteristic of depression among OA have been explicitly associated with corresponding changes in affect. For example, positive and negative life events are associated with increases in positive affect and negative affect respectively [34]. From the abnormal psychology literature, depression has historically been conceptualized as an affectively complex syndrome that is best differentiated from anxiety by assessing for the presence of low positive affect, or anhedonia [35,36]. This conceptualization was extended by the ‘tripartite model’, which conceptualized depression and anxiety using a three-factor model of nonspecific-general distress, low positive affect, and somatic arousal [37]. Importantly, the tripartite model has been demonstrated to be age-invariant, suggesting that the decomposition of depression and anxiety into its affective components is appropriate for older adults [38]. Among OA, low positive affect has been shown to be as robust a predictor of depression as negative affect, thus suggesting that the assessment of depression in OA should include a comprehensive assessment of affect in addition to determining whether diagnostic criteria for depression has been met [39].

### Affect, depression, and cognition in OA

Although it has been established that depression is associated with cognitive deficits and that depression can be decomposed into affective components in OA, research on the neuropsychological correlates of affect in old age is scarce. With respect to ‘normal’ (i.e. non-pathological) mood, one review found that the induction of negative affect did not appear to have consistent effects on cognition whereas the induction of positive affect results in poorer memory and executive functioning performance [40]. However, it is questionable whether the experimental literature on affect and cognition is generalizable to subthreshold and threshold geriatric depression and its cognitive consequences. Two studies of healthy older adults found that increased positive affect was related to better performance on tasks of immediate recall and delayed recall [41,42]. Hill et al., [42] did not observe an association between negative affect and recognition or recall. However, Allen et al., [42] found that negative affect, as measured by the neuroticism scale of the NEO-PI-S, partially mediated the relationship between age and learning slopes across a group of episodic memory tasks.

Research that models the associations of affect, depressive symptoms, and cognitive measures simultaneously in OA is also scarce. A number of US-based studies of older adults have modeled similar associations between affect, depressive symptoms, and cognitive ability across multiple domains. One longitudinal study of African American OA used the total and affect facet scores of the Center for Epidemiologic Studies Depression Scale-Revised (CES-D) and the Geriatric Depression Scale (GDS) to study the differential effects of total scores and facet scores on measures of mental status and multiple cognitive domains. The study found that the total score for the CES-D was unrelated to all cognitive measures except mental status while the total score for the GDS was significantly associated with accelerated rates cognitive decline on measures of mental status, semantic memory, and working memory. With regard to the affect facets, the anhedonia/ low positive affect facets of the CES-D and the GDS were related to declines in episodic memory and perceptual speed and declines in semantic memory respectively. Negative affect and somatic complaints were unrelated to any cognitive measures for the CES-D while negative affect on the GDS was associated with declining mental status [43] A longitudinal study of women older than 65 at increased risk for breast cancer used the Positive and Negative Affect Scale (PANAS) and the GDS to study the effects of negative and positive affect and depressive symptoms on cognition across multiple domains. The study found that higher scores on the GDS were associated with poorer performance on measures of memory and verbal knowledge. Positive affect and negative affect were associated with better letter and category fluency and poorer global cognition and poorer visuospatial ability respectively [44]. Using factor analytically derived facets of the GDS, Hall et al., [45] found that the cognitive impairment facet was inversely associated with visual memory and recall, verbal memory and recall, and switching and the apathy facet associated inversely with attention for male and female healthy OA. In this sample, the meaninglessness subscale predicted poorer verbal fluency for females while there were no significant predictors of verbal fluency for males. In a study by the same group, the pattern of associations between the GDS facets and the Repeatable Battery for Neuropsychological Status (RBANS) subtests showed that meaninglessness and dysphoria were robust predictors of multiple domains of cognitive functioning, but that the observed effects were larger for Hispanics relative to non-Hispanics [46]. The emerging research suggests that depressive symptoms and negative and positive affect likely make unique contributions to cognitive decline in OA. However, the cognitive domains associated with measures of depressive symptomatology and affect do not appear to be consistent. The goal of the present study was to extend these models reviewed here to include Latin Americans in Costa Rica.

### Goal of the present study

The goal of the present study was to extend the existing research on depression, affect, and cognition to a sample of community-dwelling Costa Rican OA. Using structural equation modeling (SEM), we tested the hypothesis that latent variables analysis would be sensitive to increased Negative Affect and Geriatric Depression and decreased Positive Affect would predict poorer performance on latent measurements of Verbal Memory, Verbal Reasoning, Processing Speed, and Working Memory after controlling Age, Education, and self-rated Health.

## Methods

### Participants

Participants for this study were 184 older adults. Participants were recruited over a 2-year period and were volunteers recruited from programs in San Jose, Costa Rica offering educational, recreation, and social participation for older adults that are overseen by organizations including Costa Rica Gerontological Association (Asociación Gerontológica Costarricense, AGECO) and the Integral Program for Older Adults from the University of Costa Rica (Programa Integral para Personas Adultas Mayores, PIAM). To meet inclusion criteria, subjects had to be ages 65–85 and score in the unimpaired range on the cognitive status screen (MMSE >24). Participants had to have visual and auditory abilities sufficient to complete all cognitive assessments and to have been on stable doses of medications for at least 30 days prior to screening. Participants with a history of clinically evident stroke or clinically significant infection within the past 30 days were also excluded. Participants with current clinically significant systemic illness or significant pain or musculoskeletal disorder that would prohibit participation in fitness testing were excluded.

Participants meeting criteria for clinically significant psychiatric disorder according to *DSM-IV* criteria or experiencing significant psychiatric symptoms (e.g. hallucinations) were excluded. Participants with a history of alcohol or drug abuse or dependence within two years were also excluded.

### Measures of depression and affect

#### Geriatric depression scale (GDS)

The GDS is a 30-item measure designed to assess the psychological dimensions of depression in OA with depressive symptomatology [47]. The scale features dichotomized response options (yes/no) and asks participants to consider the previous two weeks while filling out the questionnaire. The GDS has also demonstrated acceptable sensitivity and specificity as a depression-screening tool and was shown to have equivalent criterion validity to the CES-D [48]. A version of the GDS validated for Costa Rican older adults was used in the present study [49].

#### Positive affect and negative affect scale (PANAS)

The Positive and Negative Affect Schedule (PANAS) is a 20-item assessment of positive and negative affect. Positive affect refers to perceived energy level, and “pleasurable engagement with the environment” ([36], p. 347). Negative affect refers a range of negative moods including fear, general distress, anger, and resentment ([36], p. 347). Participants were asked to indicate the extent to which they endorsed a particular mood item on a 5-point Likert scale (1=very slightly to 5=extremely) [50]. The present study used a version of the PANAS validated for Costa Rican older adults [51].

### Cognitive measures

#### Cambridge cognitive examination-R

(CAMCOG) The CAMCOG-R is the cognitive assessment section of the CAMDEX-R [52]. A Spanish translation of the CAMCOG was used for the administration of the measure in the present study [53]. The specific items from the CAMCOG-R used included the present study included the Abstract Thinking subscale, the Attention and The Calculation subscale, and the New Learning measure from the Memory subscale. The Abstract Thinking subscale is comprised of a verbal test of similarities, which assesses the ability to draw conceptual relationships between objects and general abilities of abstraction. The subtests of the Attention and Calculation subscale include serial sevens, counting backwards calculation, copying, and drawing [52]. The New Learning measure on the Memory subscale assesses incidental and intentional learning of non-verbal stimuli [52].

#### Consortium to establish a registry for alzheimer’s disease neuropsychological assessment battery (CERAD [54])

The CERAD is a standardized comprehensive assessment battery for the evaluation and diagnosis of Alzheimer’s disease [55]. The official translation was downloaded at http://cerad.mc.duke.edu. Select subtests administered here included Verbal Fluency, World List Learning - Immediate and Delayed. In verbal fluency the participant must say as many words as possible from a category in 60 seconds. The word list learning task consisted of 3-trials where the participant was read a list of 10-words aloud and then asked to recall as many as remembered. After a 15-minute delay the participant was asked to recall the list of 10-words again. The total number of words recalled at the immediate and the delay were recorded.

#### Trail making test (TMT, [56])

The TMT is a measure of simple attention, processing speed, and set switching. For Trails A, subjects connect numbered circles as quickly as possible. For Trails B, subjects alternate connecting circles with numbers and letters in order as quickly as possible. The time to complete each test, in seconds, was the measure of interest in the present study.

#### Digit span (DS) Test Wechsler adult intelligence scale-revised [57]

The DS is a measure of attention and verbal working memory. DS Forward involves reciting back a list of numbers to read to the subject. DS Backward involves reciting back a list lost of numbers in reverse order read to the subject.

### Covariates

**Figure 1:**
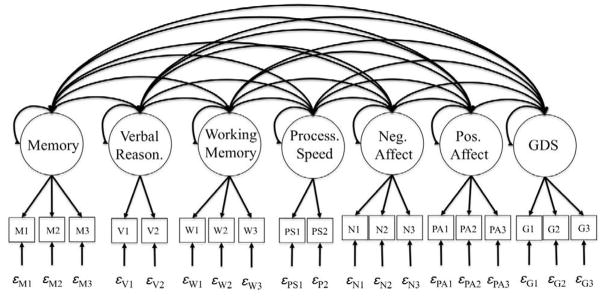
Specification of measurement model. Note: Memory: CAMCOG: total learning (M1), CERAD: total word list recall (M2), CERAD: total world list memory (M3) Verbal reasoning: CERAD: total verbal fluency (V1), CAMCOG total abstract thinking (V2) Working memory: CAMCOG: Attention/Calculation (W1), DS Forward (W2), DS Backward (W3) Processing Speed: Trails A completion time (PS1), Trails B completion time (PS2) Negative Affect: PANAS negative affect parcels, 1–3 (N1–N3) Positive Affect: PANAS positive affect parcels, 1–3 (PA1–PA3) GDS: GDS parcels, 1–3 (G1–G3). Note: Circles depict latent variables. Squares depict indicators. Epsilons represent indicator specific error variances. Single-headed arrows from latent variable to indicators represent (factor) loadings. Double headed arrows represent covariances when between latent variables and variance for single latent variables.

In all SEM analyses, all latent variables are regressed on age, education, and self-rated health (not pictured in Figure 1). The effect of gender was analyzed separately through invariance testing of the measurement model [58,59]. Tests of invariance were tested on the measurement model to test for the effects of gender [58,59]. The establishment of measurement invariance demonstrates group equivalence for model parameters at the measurement level (i.e. intercepts and factor loadings) and at the latent variable level (i.e, latent variable variances, means, and covariances). The demonstration of invariance is a necessary pre-condition to collapsing across sexes for pooled analyses.

### Proposed model

**Figure 2:**
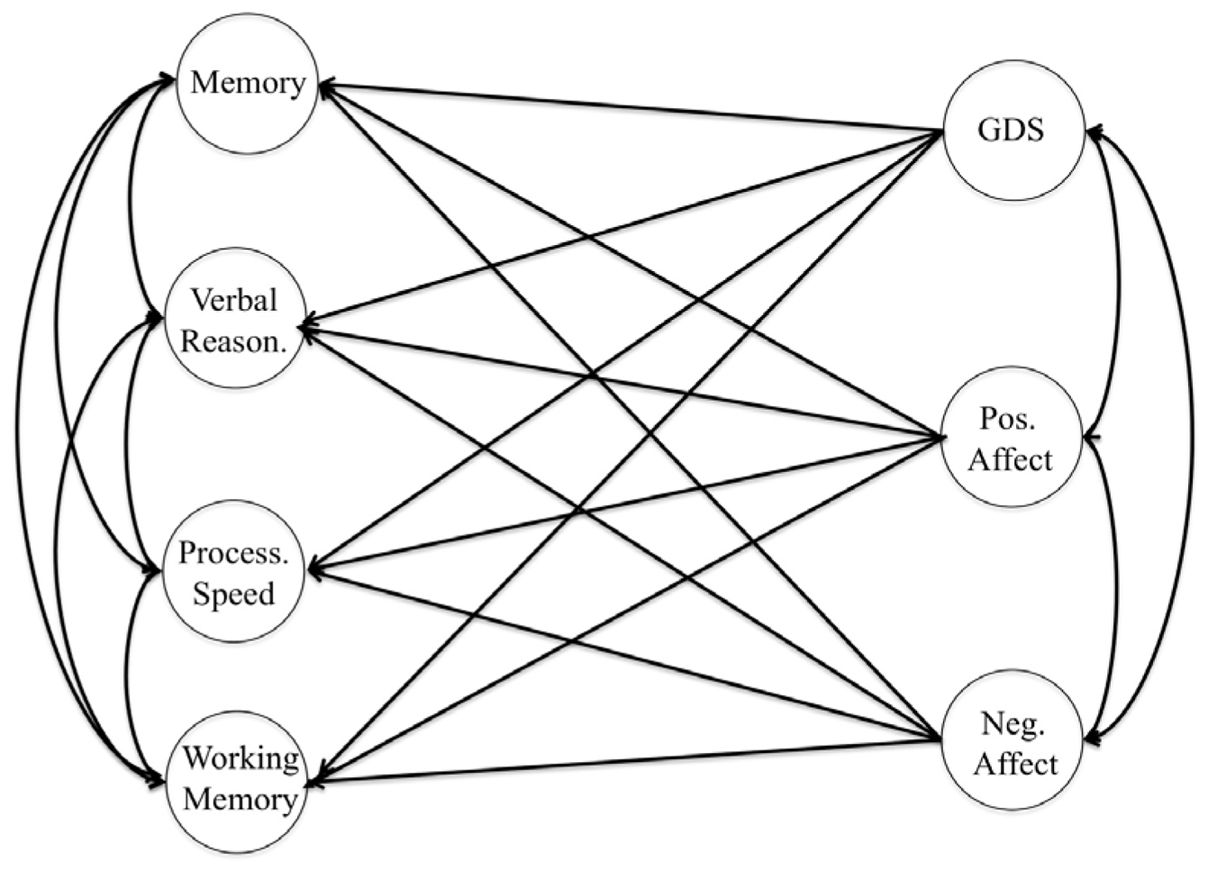
Hypothesized structural model. Circles represent latent variables. Single-headed arrows represent regression paths. Double-headed arrows represent latent covariances. Covariate effects, latent variances, and the measurement model are omitted from diagram.

We used confirmatory factor analysis (CFA) and structural equation modeling (SEM) to define and test complex relationships among a large number of tests and latent variables (factors). CFA is a common method used to aggregate true score (common) variance across multiple subtests and attenuate error. Thus, factor scores yield more reliable and sensitive estimates of cognitive ability than do traditional analyses of raw scores or scaled score composites because they are purer indices of ability [55]. Using CFA we generated 4 cognitive outcomes and 3 affective outcomes, comprising the measurement model (Figure 1). The measurement model included seven latent variables: Verbal memory (CERAD total verbal learning score, the CERAD total word recall, and the CAMCOG total learning score), Verbal Reasoning (CERAD verbal fluency score and the CAMCOG Abstract Thinking scores), Working Memory (CAMCOG Attention/Calculation subscale score, the DS backward, and DS forward scores), Processing Speed (times to completion for the Trails A and Trails B), Negative Affect, Positive Affect, and Depression. The Negative Affect, positive Affect, and Geriatric Depression were made up of parcels derived from the items comprising the items of the negative affect subscale of the PANAS, the positive affect subscale of the PANAS, and the GDS, respectively. Using SEM we tested hypothesized relationships (structural model) between affective and cognitive constructs (Figure 2).

### Analysis

The present study used CFA and SEM to model the hypothesized relations among affect, depression, and cognition and the covariates. The use of CFA and SEM allows for the modeling of true score variance by attenuating measurement error. Given the large number of indicators from the PANAS and GDS and the large number of parameters required to model them in CFA, parceling was used to conserve degrees of freedom and thus increase model parsimony. Parceling is an item aggregation method for creating parsimonious and just-identified CFA models [58,60]. Parceling has psychometric benefits in addition to parsimony such as improved reliability and closer approximations of multivariate normality [60]. Parceling is an appropriate method when the focus of study is on the structural model rather than the measurement model. We parceled the items by counter balancing the items based on their factor loadings in an initial model [60]. For the GDS, we used factor scores in order to form a more parsimonious construct and to have continuous indicators of the depression construct [61]. The factor scores were estimated from a model that deals with the binary nature of the items properly by using the WLS estimator with robust chi-square and standard errors [62–65]. The original items were grouped together into three factors scores, this factorial structure were based on an exploratory factor analysis that forced the indicators to 3 correlated factors. Full information maximum likelihood estimation (FIML, [66]) was implemented to handle missing data.

The empirical validity of each model (i.e, how well the hypothesized model fits the observed data) was assessed using goodness-of-fit indices [67]. Model selection and evaluation was primarily based on differences in the root mean square error of approximation (RMSEA, [68]), the comparative fit index (CFI, [69]), the Tucker-Lewis index (TLI, [69]) and gamma-hat (γ̂) [70]. The RMSEA indicates the degree of mismatch between the sample variance-covariance matrix and the model-implied variance-covariance matrix with acceptable values being less than 0.10 [70]. The CFI and TLI are incremental fit indices that assess the extent to which the specified model improves fit over the null model, with acceptable values approaching one [70,71]. Lastly, γ̂ is a goodness of fit measure that has been found to yield unbiased estimates of fit in small samples, with values approaching one indicating better fit [70].

The SEM analysis first tested a full model, which simultaneously regressed all cognitive latent variables on all affect and depression variables, resulting in 12 estimated regression paths. In addition, this model regressed all latent variables on all covariates, resulting in 21 estimated regression paths. After testing the full model, the statistical significance of the contribution of each regression path was tested one at a time. This was done using iterative chi-square difference tests, where a model was estimated with one regression path constrained to zero and was then compared to the full model. Paths were retained when the chi-square difference, per one degree of freedom, exceeded the critical chi-square value, when *α*=0.01. Thus, not estimating parameters that fulfilled this criterion resulted in a significant loss in absolute fit and thus warranted their inclusion in the final model. Parameters not satisfying this criterion were excluded from the final model. This trimming methodology has been used by methodologists and is a robust alternative to trimming based upon the statistical significance of individual regression coefficients [72,73]. Analyses were performed with the package Latent Variable Analysis (lavaan) released 0.5.18 [74] for the software R release 3.2.1 (R Core Team, 2015).

## Results

Table 1 presents the characteristics of the study sample. The sample was moderately old and included a wide range of ages (54–88 years old). The sample had an average level of education of 12.3 years and was predominately female (58%). With regard to the affect variables, the average level of depression as indicated on the GDS was 5.26 out of a possible 30, indicating relatively low levels of depressive symptomatology in the sample. The sample of older adults indicated relatively low levels of negative affect on average (21.97/50) and high levels of positive affect (40.64/50).

The summary statistics for the subscales of the CAMCOG and CERAD used in the present study are listed in Table 1. The scores on the CAMCOG from the present sample were slightly lower than those obtained in a large population study in England that derived norms for the CAMCOG [75]. Median scores on the Abstract Thinking subscale and on the Learning subtest were lower in the present sample (Median_Abstract Thinking_=5; Median_Learning_ =13) than the scores observed in the normative sample of non-demented British OA (Median_Abstract Thinking_=7; Median_Learning_=14) [75]. Comparison of the CAMCOG Attention/Calculation subscale from the present study with the study by Williams et al., [75] was not possible because the normative study did not derive the subscale score using all of the items from the scale. Scores on Word List Memory and Word List recall from the present study were comparable to the mean scores obtained on these tests in a normative sample of cognitively normal Brazilian OA (Word List Memory=18 (4.1); Word List Recall=5.5 (2.2) [76]. The mean verbal fluency observed in the present sample exceeded that observed by Bertolucci et al., [76] (Verbal Fluency=19.6 (4.96)).

Table 2 presents the bivariate correlation matrix for the variables and covariates used in the present analysis. Consistent with this present hypothesis, increased ratings of depressive symptoms on the GDS was associated with slower Trails B completion time, reduced verbal fluency, reduced attention/calculation ability, reduced abstract thinking, and fewer digits recalled correctly on DS backwards. The bivariate correlations of the GDS with the neuropsychological measures in this study were inconsistent in that none of the measures of memory used or measures of simple attention/processing speed (Trails A) were correlated with the GDS. In addition, the magnitude of these correlations was small (|*r*|<0.30). Interestingly, the negative affect subscale of the PANAS was not significantly correlated with any of the neuropsychological measures. This suggests that the variance shared by the GDS and the negative affect scale of the PANAS (*r*=0.56, *p<*0.05) is distinct from that shared by the GDS and the neuropsychological measures. Increased positive affect was weakly associated with verbal fluency and the CAMCOG Attention/Calculation subscale only (*r*s=0.18, *p*<0.05).

Among the neuropsychological measures, the memory measures had the highest intercorrelations (*r*s=0.50–0.7, *p<*0.05) followed by the processing speed measures. The intercorrelations among the variables comprising both the verbal ability and working memory latent variables had similar patterns of correlations and magnitudes of correlation. Digit Span forward was an exception to pattern as it failed to correlate significantly with any of the other variables.

The correlations of the covariates with the cognition and affect variables showed that age generally had a deleterious effect on across all cognitive domains assessed while education had a protective effect. Gender had no appreciable effect on any of the cognitive variables. Self-rated health was the only covariate significantly associated with measures of affect. Better self-rated health was associated with fewer depressive symptoms on the GDS, less negative affect, and with increased positive affect.

The measurement model demonstrated good fit (*χ*^2^ (133)=206.9, RMSEA=0.05 [0.04–0.07], CFI=0.9, TLI=0.94). All indicators loaded significantly onto their target latent variables. However, DS forwards loaded marginally onto the working memory latent variable (*λ*=0.24 (0.10), *p*=0.02). The small magnitude of this loading was discrepant with the loadings of the other indicators on the working memory latent variable (*λ*_CAMCOG-A/C=_0.51 (0.06), *p<*0.01; *λ*_DSbackwards_=0.50 (0.09), *p*<0.01). DS forward’s weak loading is likely due to the fact that DS forward taps the maintenance component of working memory and does not require participants to manipulate information in mind as DS backwards and the subtests comprising the attention/calculation facet of the CAMCOG do. Invariance testing for the effect of gender on the model demonstrated equivalent indicator loadings, intercepts, latent variable means, latent variable variances, and latent covariances. Thus, collapsing across gender for subsequent analyses was warranted.

The hypothesized full SEM model is displayed in Figure 2. Model fit for the full SEM model was good (*χ*^2^ (169)=245, RMSEA=0.05 [0.04–0.06], CFI=0.96, TLI=0.9, γ̂ =0.96). Results of the full model indicated that the only significant regression paths among the latent variables was for the regression of working memory on depression (*β*=−0.49 (0.25), *p*<0.01). In addition, after adjusting positive and negative affect for age, education, and self-reported health, positive and negative affect were no longer significantly correlated (*r=*−0.1, *p=*0.29). Significant covariate effects were found for memory on age (*β*= −0.40 (0.14), *p*<0.01), the effect of processing speed on age (*β*=0.32 (0.15), *p*<0.01) and education (*β*= −0.44 (0.03), *p*<0.01). Poorer verbal reasoning was associated with older age (*β*= −0.36 (0.02), *p*<0.01) and fewer years of education (*β*=0.31 (0.03), *p*<0.01). Better working memory was associated with increased education (*β*=0.43 (0.04), *p*<0.01). Depression and negative affect were associated with decreased self-rated health whereas positive affect was positively associated with self-rated health.

**Figure 3:**
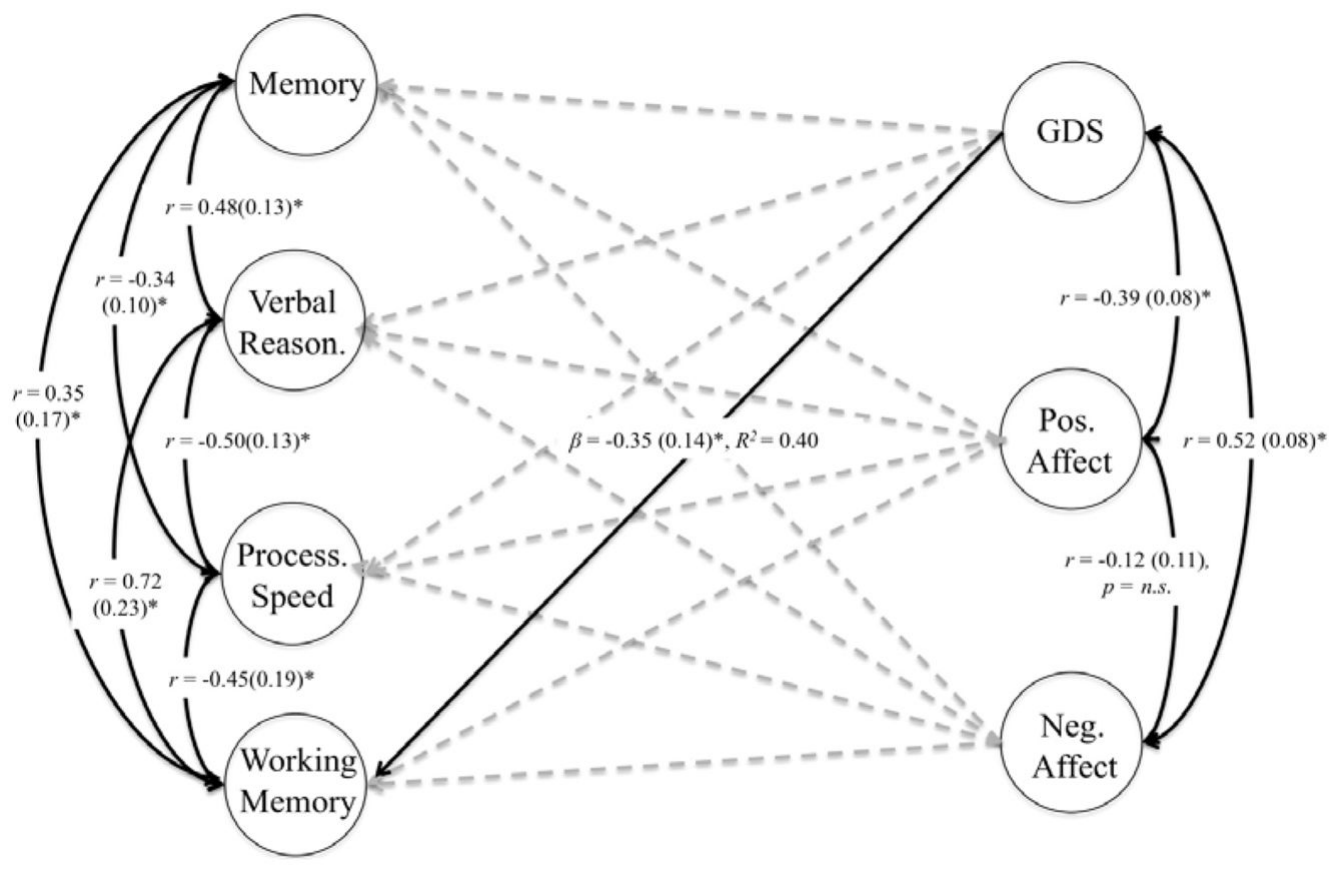
Results for the final structural model. Grey dashed paths were non-significant in initial model and constrained to equal zero. Note: Fit for trimmed model: χ2 (191)=273.67, RMSEA=0.05 [0.04–0.06], CFI=0.95, TLI=0.94, γ̂=0.96 *p<0.05.

Iterative chi-square difference testing revealed that models constraining covariate effects to zero including memory on age, attention on age and education, depression on self-rated health, positive affect on self-rated health and age, negative affect on self-rate health and age, verbal reasoning on age and education, and working memory on education resulted in significant decrements in model fit and were thus included in the final model. The effect of working memory on depression was the only non-covariate path to result in significant decrements in fit when constrained and thus was included in the final model (Δ*χ*^2^ (1)=14.07, *p<*0.001). The trimmed model resulted in no significant loss of model fit relative to the full model (Δ*χ*^2^ (22)=30.3, *p=*0.11) and did not appreciably change any of the other fit indices. Parameter estimates for the Final Model are displayed in Figure 3. The final model revealed that increased depression significantly predicted decreased working memory capacity (*β*= −0.31 (0.14), *p*<0.01). Of the covariate effects, all were comparable to those estimated in the full model, with the exception of the inverse effect of negative affect on age (*β*= −0.14 (0.02), *p*=0.03), which was non-significant in the full model.

## Discussion

We tested a large sample of healthy aging Hispanic-Central Americans using a broad neuropsychological battery that sampled both cognitive and affective states including Verbal Memory, Verbal Reasoning, Processing Speed, Working Memory, Positive Affect, Negative Affect and Geriatric Depression. In general, scores on the cognitive measures were comparable to age and education normative data for lower educated convenience samples used widely in US clinical research [77–80]. These Costa Rican’s differed from US-based sample in their self-reported affective profiles. Positive affect was very high, 10 points higher than US normed comparisons (31.3 vs. 40.6, out of 50 possible) while negative affect was about equivalent [81]. Thus these Costa Rican older adults were happy, as evidenced by the high ratio of positive affect to relatively low negative affect. Similar to their negative affect scores, these older adults’ geriatric depression scores were also low but not substantively different than US-based healthy aging community samples [81]. Given this sample’s relatively good health, average negative affect/depressive symptoms, and high positive affect, we tested the role of affect and depression on cognitive performance using a standard battery of neuropsychological measures.

Costa Ricans have been found to be one of the happiest nations in the world in the survey literature [http://www.well-beingindex.com/]. By result we believed that their affective profiles would differ substantively from common US affective profiles and also be differentially related to their cognitive performance. We used SEM to understand if positive affect plays a greater role in the cognitive-affective profile of Costa Rican’s than most US studies that find broad cognitive decrements, especially depression [19]. We were somewhat surprised to find that depressive symptoms were selectively correlated to decrements in working memory and that negative and positive affect contributed very limited amounts of variance to any of the cognitive factors. In hindsight, these results are fully convergent with the findings of a large and well-controlled meta-analysis of the neuropsychological impact of depression on cognitive ability [19] that showed that executive function, especially working memory tasks, were most susceptible to depression. Our results are the most frequently occurring when power is sufficient and methods rigorous. Parallel findings in US-based samples include a study by Turner et al., [43], which found an inverse relationship between depressive symptoms indicated on the GDS and working memory ability was observed. In addition, O’Bryant et al., [46] observed and inverse association between depression, as measured by the GDS, and attention. Given that working memory tasks also involve attentional processes, these findings are germane to the findings in the present study. However, the results reported by Danhauer et al., [44] were discrepant in that depression failed to predict working memory performance in this study. US-based studies examining the effect of depressive symptoms and negative and positive affect have observed negative effects of GDS symptoms on measures of semantic memory, verbal memory, immediate recall, delayed recall, figural memory, and visuospatial ability [43,44,46].

The current SEM has unique analytical strengths. First, application of CFA is a multivariate measurement of common variance that attenuates error variance and results in purer indices of each factor. Second, the use of SEM allowed us to model explicitly the covariance among cognitive factors and among affective measures. Thus, we were able to understand each factor’s unique contribution to the model, given its interdependence on the other latent variables. The reported results are very specific. This WM factor is not contaminated with speed of processing or other measured cognitive factors (cf, [19] for comment on depressive effects on WM in the absence of psychomotor retardation). Likewise, the GDS represents symptoms that are richly cognitive - free of negative or positive affect. Thus, the current model suggests that working memory buffer size - not speed - was particularly susceptible to the effects of cognitive symptomatology measured by the GDS. Core depressive symptoms that affect cognition appear to be uniquely related to cognitive dysfunction over and above any individual’s predisposition toward negative or low positive emotional components.

The results from the present study did not observe associations between depressive symptoms and memory or reasoning abilities (neither zero-order correlations nor structural paths tested in SEM). High or low levels of affect did not affect cognition. The lack of an association of positive or negative affect with any of the cognitive latent variables diverged from the findings of other studies [43,44]. Our study methodology was well-controlled and our sample is likely to be different from these US-based samples. Costa Rican older adults experience more positive affect and equivalent negative affect and thus the ratio of positive to negative may help explain this difference. High positive affect is likely to dampen the impact of negative affect on cognition [36]. One could imagine that our Costa Rican older adults are emotionally more stable and thus less distractible.

Though this report is based on a convenience sample, these Costa Rican older adults benchmark successful aging among Hispanics-Central Americans. They provide us some insight into what Hispanic aging looks like in the absence of the sociopolitical pressures that mark so much of US health disparities research on US Latino aging. While this paper provides cognitive aging researchers a touchstone of healthy Hispanic aging, it also greatly limits the generalizability of these findings. Use of these data as norms for Hispanic-US Americans who live in the current US political landscape would be inappropriate.

Future research on the cognitive consequences of depression in Latin American older adults should aim to use to longitudinal research designs and sample from multiple Latin American nations in order to better understand differences in cognitive aging in Latin America.

## Figures and Tables

**Table 1:** Sample Characteristics (N=184).

	Range	Mean (*SD*)
Age	54–88	68.49 (7.74)
Years of Education	2–28	12.27 4.83)
Female (F/M)	-	107/77
MMSE	25–30	28.56 (1.54)
Self-Rated Health	1–5	3.98 (0.95)
Total GDS, mean	0–25	5.26 (5.03)
Total Negative Affect	10–45	21.97 (7.75)
Total Positive Affect	18–50	40.64 (6.29)
**Verbal reasoning**		
CERAD: Total Verbal Fluency	9–31	19.57 (4.62)
CAMCOG: Total Abstract Thinking	0–8	4.82 (2.23)
**Memory**		
CAMCOG: Total Learning	5–17	12.90 (2.47)
CERAD: Total Word List Recall	0–10	5.44 (2.08)
CERAD: Total Word List Memory	6–26	16.13 (3.87)
**Working memory**		
CAMCOG: Total Attention & Calculation	2–9	8.15 (1.36)
Digit Span Forwards	3–9	5.32 (1.07)
Digit Span Backwards	2–6	3.56 (0.98)
**Processing speed**		
Trail Making Test A (completion time)	20–167	66.42 (25.80)
Trail Making Test B (completion time)	40–573	158.03 (89.26)

**Table 2:** Variable bivariate correlations.

	2	3	4	5	6	7	8	9	10	11	12	13	14	15	16	17
1. Age	−0.06	−**0.38**	−0.09	0.04	−0.08	−0.08	−**0.29**	−**0.35**	−**0.41**	**0.40**	**0.38**	−**0.36**	−**0.20**	−**0.3**	0.02	−**0.17**
2. Gender	1	**0.19**	**0.15**	−0.07	−0.09	0.01	−0.06	−0.02	−0.04	−0.05	−0.04	0.01	0.17	−0.06	−0.02	−0.01
3. Education		1	**0.19**	−**0.17**	0.11	0	**0.19**	0.14	**0.25**	−**0.47**	−**0.44**	**0.31**	**0.28**	**0.33**	**0.16**	**0.27**
4. Self-Rated Health			1	−**0.48**	**0.3**	−**0.39**	**0.17**	0.12	**0.15**	−0.08	−0.09	**0.19**	0.11	0.10	0.10	**0.15**
5. GDS				1	−**0.4**	**0.56**	−0.11	−0.1	−0.11	0.06	**0.16**	−**0.17**	−**0.18**	−**0.16**	−0.04	−**0.21**
6. PANAS positive					1	−**0.19**	−0.04	0.11	−0.03	−0.03	−0.01	**0.18**	**0.18**	0.04	0.08	0.06
7. PANAS negative						1	−0.09	0.02	−0.11	−0.05	0.01	0.04	−0.02	0.01	0.12	−0.06
8. CERAD: Total Wordlist Memory							1	**0.5**	**0.73**	−**0.32**	−**0.23**	**0.29**	**0.21**	**0.31**	0.04	**0.26**
9. CAMCOG: Total Learning								1	**0.5**	−**0.31**	−**0.37**	**0.33**	**0.22**	**0.32**	−0.03	0.09
10. CERAD: Total Wordlist Recall									1	−**0.38**	−**0.28**	**0.29**	**0.21**	**0.32**	−0.08	**0.2**
11. TMT A Completion Time										1	**0.57**	−**0.35**	−**0.22**	−**0.38**	−0.12	−**0.26**
12. TMT B Completion Time											1	−**0.33**	−**0.3**	−**0.35**	−0.1	−**0.23**
13. Verbal Fluency												1	**0.29**	**0.38**	0.13	**0.32**
14. CAMCOG Attention/Calc.													1	**0.18**	0.15	**0.21**
15. CAMCOG Abstract Thinking														1	0.04	**0.18**
16. DS Forwards															1	**0.18**
17. DS Backwards																**1**

* *Note: bold values, p*<0.05
